# A digital photography dataset for Vaccinia Virus plaque quantification using Deep Learning

**DOI:** 10.1038/s41597-025-05030-8

**Published:** 2025-04-30

**Authors:** Trina De, Subasini Thangamani, Adrian Urbański, Artur Yakimovich

**Affiliations:** 1 Center for Advanced Systems Understanding (CASUS), Görlitz, 02826 Germany; 2 Helmholtz-Zentrum Dresden-Rossendorf e. V. (HZDR), Dresden, 01328 Germany; 3 Department of Computer Science, Technische Universität Dresden, Dresden, 01069 Germany; 4 Institute of Computer Science, University of Wrocław, Wrocław, 50-383 Poland

**Keywords:** Microbiology, Image processing, Machine learning

## Abstract

Virological plaque assay is the major method of detecting and quantifying infectious viruses in research and diagnostic samples. Furthermore, viral plaque phenotypes contain information about the life cycle and spreading mechanism of the virus forming them. While some modernisations have been proposed, the conventional assay typically involves manual quantification of plaque phenotypes, which is both laborious and time-consuming. Here, we present an annotated dataset of digital photographs of plaque assay plates of Vaccinia virus - a prototypic propoxvirus. We demonstrate how analysis of these plates can be performed using deep learning by training models based on the leading architecture for biomedical instance segmentation - StarDist. Finally, we show that the entire analysis can be achieved in a single step by HydraStarDist - the modified architecture we propose.

## Background & Summary

Poxviruses are large DNA viruses that infect a wide variety of vertebrates including humans causing a range of diseases^1^. Most notably smallpox, caused by the variola virus, is known for a great number of deadly outbreaks devastating human populations up until its eradication in the 20th century^2^. Yet, despite smallpox eradication, poxviruses continue to present a real-world healthcare challenge^3,4^. Recently, a global monkeypox outbreak caused a global healthcare emergency directly demonstrating the importance of continued poxvirus research^5^.

One vital instrument in conducting poxvirus research in a laboratory is the ability to quantify infectious virus particles in the inoculum^6–8^. The assay involves infecting a monolayer of host cells (indicator cells) with varying concentrations of a highly diluted virus inoculum. Upon a period of incubation (e.g. 48 hours), cells are fixed with formaldehyde and stained with a dye to reveal the areas of cell death or plaques. Counting the number of plaques allows for calculating the number of infectious particles in the original inoculum^6^.

Beyond the means to quantify the infectious virus, virological plaque assay represents an experimental system allowing us to study virus replication and spread^9–11^. Yet, capturing this information often requires a modification to the original assay employing quantitative microscopy^9,11^ leading to exceeding costs in equipment and personnel. Furthermore, operating complex equipment at biosafety levels higher than 2 is cumbersome. At the same time, the conventional protocol employing fixation of the cultured infected cells crystal and violet staining remains the most widely spread across the laboratories of the world due to its simplicity. This, in turn, leads to the continued use of the classic assay combined with manual quantification.

However, recent advances in machine learning (ML) and deep learning (DL) for computer vision^12^, as well as the use of digital photography in research^13^ may provide the means necessary for a technological leap allowing to improve upon the classic assays like the plaque assay. Following this, here we present an annotated open dataset of Vaccinia virus (VACV a prototypic poxvirus) plaque assays performed in 6-well plates and captured by digital photography - VACVPlaque^14^. We validate applicability of this dataset for instance segmentation task using state-of-the-art architecture. Furthermore, we propose an improved architecture allowing for the detection and segmentation of wells and individual plaques in these photographs in a single-step end-to-end manner.

While other ML-based approaches to plaque quantification have been proposed in the past^15,16^, due to the relatively simplistic ML algorithms employed these approaches require more laborious and standardised image acquisition techniques (flatbed scanner or an apparatus assembly). In contrast, based on a convolutional neural network (CNN)^12^, our approach excels with digital photographs taken with a simple handheld mobile phone. Since one major obstacle to the adoption of DL in Infection Biology is the lack of large annotated datasets, we argue that the opening of the dataset employed in this work to the research community will further facilitate the development of advanced algorithms for virological plaque quantification.

## Methods

### Data preparation and collection

**Fig. 1:**
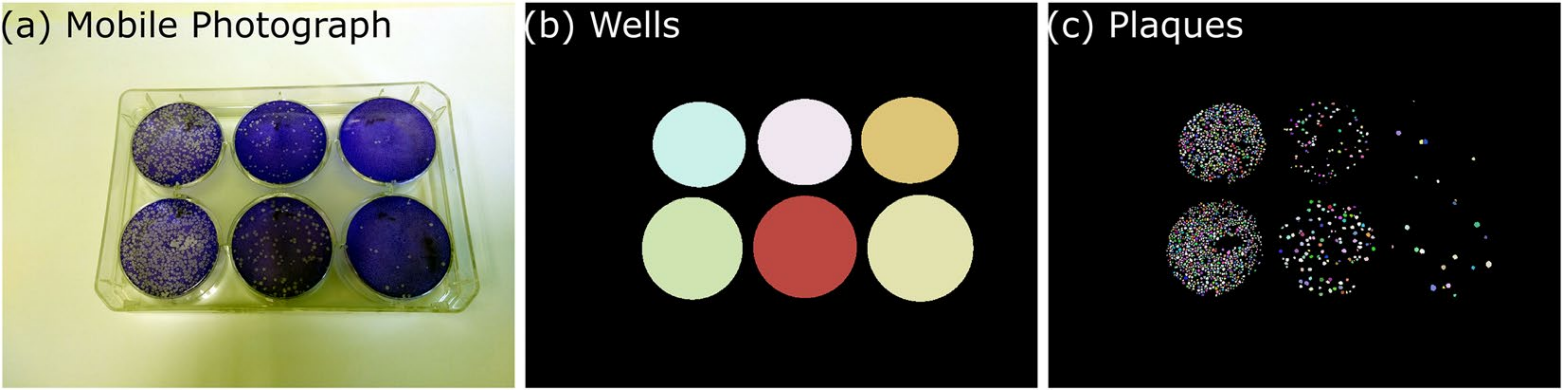
VACVPlaque^14^ example containing **(a)** RGB digital photographs of plaques within 6-well tissue culture plate, *x*_*n*_. **(b)** Annotations of *x*_*n*_ corresponding to the wells instance mask ${Y}_{n}^{2}$ and **(c)** plaques instance mask ${Y}_{n}^{1}$. The well diameter is 35 mm for scale.

The dataset is derived from 211 digital photographs of 6-well tissue culture plates (Fig. 1a), in which the VACV Western Reserve plaque assay was performed. Two digital photography devices, one from Apple Inc. (an iPhone 6) and one from Xiaomi Inc. (a Mi A1), were used to collect this dataset, and also two different perspectives of some plates were captured to form a total of 211 distinct images. Technical details as to the setup for the digital image capture can be found in Table 1.

**Table 1:** Digital Photography capture settings from the two devices.

Setting	iPhone 16	Mi A1
Make	Apple	Xiaomi
Software Version	11.4.1	tissot-user 8.1.0
		OPM1.171019.026
		V9.6.7.0.ODHMIFE
		release-keys
Orientation	6 (Rotated 90^∘^ CCW)	1 (Normal)
Resolution X (inches)	72	72
Resolution Y (inches)	72	72
Pixel Height	2448	3000
Pixel Width	3264	4000
Colour Model	RGB	RGB
Depth	8	8
Colour Profile	sRGB IEC61966-2.1	sRGB IEC61966-2.1
Aperture	2,275	2,270
Brightness	4,213	1,150
Exposure Mode	Auto	Auto
Exposure Program	Normal program	Not defined
Exposure Bias Value	0	—
Exposure Time	$\frac{1}{33}$	$\frac{1}{50}$
ISO Speed Ratings	50	400
Flash	off	off
Metering Mode	Pattern	Centre Weighted Average
FNumber	2,2	2,2
Focal Length	4,15	3,81
Focal Length (35 mm Film)	29	26
Scene Capture Type	Standard	Standard
Sensing Method	One-chip colour area sensor	One-chip colour area sensor
ISO Speed Ratings	50	400
Shutter Speed	$\frac{1}{35}$	$\frac{1}{50}$
White Balance	Auto	Auto

*Setting* indicates the name of the setting and the values corresponding to the setting for the two devices used can be found under *iPhone 16* and *Mi A1*.

Plaque assays were performed as outlined in^17^. Specifically, cultured BSC40 cells (ATCC) were seeded in 6-well plates in DMEM medium (10% fetal bovine serum (FBS, Sigma), 2 mM GlutaMAX (Life Technologies), and 1% penicillin-streptomycin (Pen-Strep, Sigma)) at conditions to form a monolayer. Next, cells were infected by VACV Western Reserve in various experimental conditions required for the particular assay. Upon incubation (typically 48h), cells were fixed with 4% paraformaldehyde (PFA) and stained with a Crystal Violet containing solution. Finally, the excess dye was washed out of the plates. For the purpose of data collection here, respective plates were repurposed from a variety of experiments, most of which involved virus titration, as well as virus titration in the presence or absence of a VACV inhibitor such as cycloheximide and AraC. This approach ensured a realistic sampling of assays from a virology lab. To capture images of repurposed experimental plates, we sequentially positioned them on a wiped laboratory bench under artificial ambient light. To diversify the condition, images were collected in several locations of the same laboratory on different days.

### Annotation

Annotation of wells in the VACVPlaque^14^ dataset was performed using a custom ImageJ/Fiji^18^ macro allowing for user-assisted allocations of the selections of the 6-well plates. Upon correction, the macro created a binary mask from the selections. Annotation of the plaques was performed manually using drawing tools of ImageJ/Fiji^18^ to create a mask in an independent layer superimposed on the RGB image (Fig. 1b,c).

### Data pre-processing

**Fig. 2:**
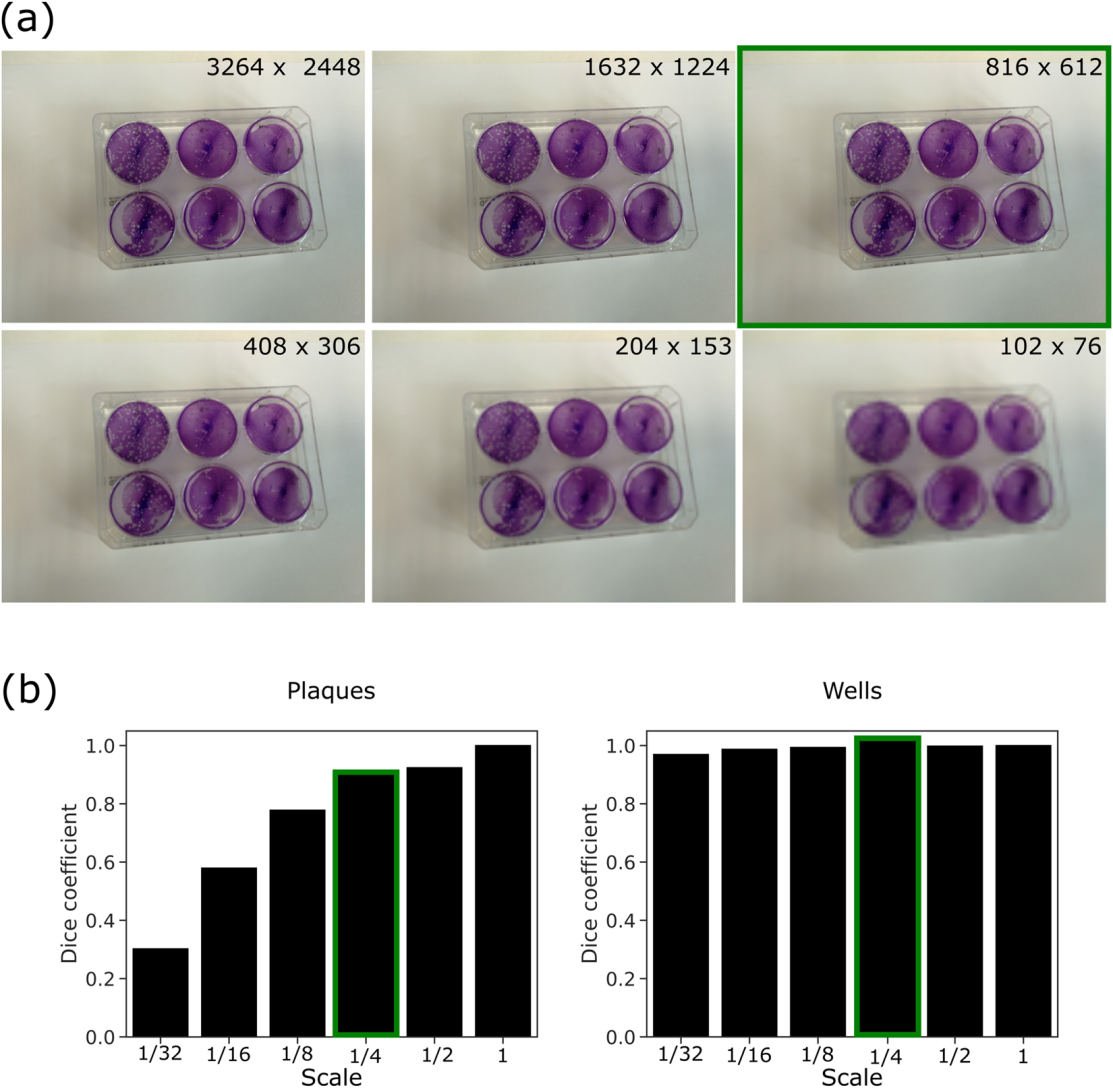
Resolution choice. **(a)** Down-scaled images at scale $1,\frac{1}{2},\,\frac{1}{4},\,\frac{1}{8},\,\frac{1}{16},\,\frac{1}{32}$ respectively in raster-scan order (left to right), **(b)** Quantification of information loss in terms of *Dice Coefficient* (Eq. (1)) for Plaques (*left*) and Wells (*right*) with scores of 0.881 and 0.997 respectively. The highlighted image and highlighted bars for the Dice Coefficient in green, are the ones corresponding to our chosen scale = $\frac{1}{4}$.

Given that each digital photograph contains nearly 8 million pixels, to minimise computational complexity during model training (see Section Technical Validation) we decided to optimise the image size to contain just enough resolution to detect the smallest plaques at $\frac{1}{4}$ of the original resolution along both the *x* and *y* axes. Figure 2, presents a detailed view of images at respective down-scaling factors for the dataset, as well as the quantification of the information loss. For VACVPlaque^14^ at $\frac{1}{4}$ of the full resolution, the values are 0.881 and 0.997 for plaques and wells respectively. The information loss in both object categories was low with the loss being higher for the smaller object (i.e. plaques). The smaller object is more likely to get lost upon down-scaling and subsequent up-scaling. Also, comparing the images at the chosen lowered resolution (highlighted in green) in panel (a) of Fig. 2 to its full resolution counter-part (left most image on the first row), we observed minimal information loss as indicated by the Dice Coefficient (Eq. (1)) values. We, therefore, concluded that the chosen resolution represents a good trade-off to making the computational costs more manageable. Noteworthy, due to the inherent properties of the DL models, this trade-off should only play a role at training time. The minimal information loss is likely acceptable, as plaques appear of different sizes depending on the proximity of the phone camera to the plate due to the plaque self-similarity. A model trained on down-scaled images should be able to pick up the smaller plaques in closer proximity at inference time.

### Quantification of information loss in down-scaling

To judge the information loss, we have used the Dice Coefficient^19,20^ given by the formula,

1 $$D=\frac{2\cdot {\sum }_{i,j}{Y}_{i,j}\cap {Y}_{i,j}^{r}+c}{{\sum }_{i,j}{Y}_{i,j}+{\sum }_{i,j}{Y}_{i,j}^{r}+c}$$

where *Y*_*i*,*j*_ and ${Y}_{i,j}^{r}$ are the binarised masks of one particular object category at full resolution and those up-scaled to full resolution from down-scale factor *r* respectively, having values in (0,1]. *c* = 1 is a smoothing factor to prevent resulting in 0 or undefined values when both of the masks are empty or there are no matching objects. This metric is lower and gives a penalty when objects in *Y*_*i*,*j*_ are lost in ${Y}_{i,j}^{r}$ due to up-scaling from a lower resolution. Figure 2 demonstrates the average value of this metric over all images for a particular down-scale factor *r*.

### DL validation training losses

The loss of SD architecture is a combination of two losses based on the two values generated by the model to predict star-convex^21^ polygons. Namely, **Object Boundary Loss** (${{\mathscr{L}}}_{OBL}$ based on the normalised Euclidean distance to the nearest background pixel *d*_*i*,*j*_) and **Distance Loss** (${{\mathscr{L}}}_{DL}$ based on ${r}_{i,j}^{k}$ the Euclidean distance to the object boundary along *k*, each of which is one of *K* equispaced radial directions around 2*π*, *k* ∈ {0, 1, 2, . . . , *K* − 1}). The total loss ${\mathscr{L}}$ is defined as:

2 $${\mathscr{L}}={{\mathscr{L}}}_{OBL}+{\lambda }_{1}\cdot {{\mathscr{L}}}_{DL},$$

where ${{\mathscr{L}}}_{OBL}$ measures the difference between the predicted boundary distance probability map and the ground truth boundary distance probability map. It encourages the model to accurately locate object boundaries using a Binary Cross-Entropy Loss (BCE)^22^ on what are probabilities representative of whether a pixel is in the interior or exterior of an object.

The ${{\mathscr{L}}}_{DL}$ quantifies the error in predicting the radial distances in the *K* equispaced radial directions. This can be formulated as a regression loss, between predicted and ground truth distances. In this case, we use Mean Absolute Error (MAE)^23^ or *L*_1_ Loss term. *λ*_1_ is a regularisation factor. This can be set to a high value to essentially prevent over or under-prediction of the distance of polygon vertices from the star centre^21,24^ along radial directions *k*.

The baseline loss effectively as concretely written in the SplineDist paper^25^can be written as,

3 $${\mathscr{L}}={{\mathscr{L}}}_{BCE}({d}_{i,j},{\hat{d}}_{i,j})+{\lambda }_{1}\cdot ({d}_{i,j}\cdot {{\bf{1}}}_{{d}_{i,j} > 0}\cdot \frac{1}{K}\mathop{\sum }\limits_{k=0}^{K-1}|{r}_{i,j}^{k}-{\hat{r}}_{i,j}^{k}|+{\lambda }_{2}\cdot {{\bf{1}}}_{{d}_{i,j}=0}\cdot \frac{1}{K}\mathop{\sum }\limits_{k=0}^{K-1}|{\hat{r}}_{i,j}^{k}|)$$

This loss is finally averaged over all the pixels in the image. Here *λ*_2_ is a regularisation term used to penalise polygon predictions around star centres where *d*_*i*,*j*_ is 0, i.e. polygon predictions centred at ground-truth background pixels. This can be set to a high value to reduce the number of such polygon predictions.

The total loss over all pixels in our proposed architecture HSD is defined as:

4 $${{\mathscr{L}}}^{{\prime} }=\sum _{i,j}{{\mathscr{L}}}_{1}+\sum _{i,j}{{\mathscr{L}}}_{2},$$

where ${{\mathscr{L}}}_{1}$ and ${{\mathscr{L}}}_{2}$ are individually the same as in Eq. (3) for each pixel for the two branched decoders respectively. Here, we penalise the prediction of plaque instances outside the boundary of wells (which according to prior knowledge should not occur). Implicitly, we also penalise errors in the prediction of the total and individual area of wells.

### Dataset validation performance evaluation metrics

To evaluate the performance in nested instance segmentation, we have used two metrics from^26^, Intersection over Union over recalled objects (*IoU*_*R*_) and Average Precision (AP) that are relevant to this task. The first metric is defined as,

5 $$Io{U}_{R}(\tau )=\sum _{{o}_{i},{\widehat{o}}_{i}\forall i\in {(O\cap \widehat{O})}_{\tau }}\frac{IoU({o}_{i},{\widehat{o}}_{i})}{| O| }$$

where *IoU* is as defined in^27^, *O* is the set of all true objects and $\widehat{O}$ is the set of all detected objects. ${(O\cap \widehat{O})}_{\tau }$ is the set of all ground truth objects identified correctly at *IoU*^27^ threshold *τ* ∈ (0, 1). ∣*O*∣ is the number of true objects. Eq. (5) can be thought of as the traditional classification performance metric Recall^28^ where we get a score of $IoU({o}_{i},{\widehat{o}}_{i})$ for each correctly recalled object instead of 1 and we normalise by TP+FN which is here ∣*O*∣.

The second metric is defined as:

6 $$AP(\tau )=\frac{T{P}_{\tau }}{T{P}_{\tau }+F{N}_{\tau }+F{P}_{\tau }},$$

for an image with regards to the two sets *O* and $\widehat{O}$ as defined above. Set theoretically, we have the membership of the above sets as follows. $o\,or\,\widehat{o}\in T{P}_{\tau }$ i.e. in $(O\cap \widehat{O})$ if its *IoU*^27^ is greater than *τ* ∈ (0, 1). Similarly we have *FN*_*τ*_ if $o\in (O\backslash \widehat{O})$ and *FP*_*τ*_ if $\widehat{o}\in (\widehat{O}\backslash O)$.\ is the set difference operator^29^. *TN*_*τ*_ is infeasible to report since there can be an infinite number of star-convex^21^ polygons that should not be detected within an image. The metric values are averaged over the images and are different for different values of *τ*.

### Dataset validation experimental setup and implementation details

In our training procedure, we ran the training from randomly initialised weights for 400 epochs for each experiment giving the model sufficient time to converge. Random patches of size (256 x 256) from the training images were used.

The skeleton of our code is the excellent StarDist repository^26^ and we modify modules where necessary to facilitate changes related to the architecture, loss computations, dataset generation and predictions. We have implemented our work in DL framework Tensorflow^30^. Based on our investigation about the reconstruction ability of our objects using such star-convex^21^ polygons, we have chosen the number of ${r}_{i\,j}^{k}$, *K* = 32 and *λ*_1_ = 0.2, *λ*_2_ = 0.0001 (see Subsection DL validation training losses) for all of our experiments. To optimise the model’s parameters, we employed the Adam optimiser^31^ with an initial learning rate of 0.0003. Then, we decreased the learning rate according to ReduceLROnPlateau^32^ with a decay rate of 0.5 with a patience of 40. The data for each epoch were random patches of size (256 x 256) from the training images with only atmost 0.1 fraction of patches containing only background pixels. We used a batch size of 32, and the model’s parameters were updated with mini-batch gradient descent^33^. The model was built in Python 3.9.18 and all experiments were conducted on a NVIDIA RTX A6000 48GB GPU device.

## Data Records

The dataset is available on RODARE^14^. It is an Open Access dataset shared under the Creative Commons Attribution 4.0 International license. The dataset is derived from 211 digital photographs of 6-well tissue culture plates, in which the VACV Western Reserve plaque assay was performed. The plaque assays were originally performed as described in^17^, used for their primary research purpose and then reused for the creation of the dataset. These plates were kindly donated by the group of Prof. Dr. Jason Mercer (University of Birmingham). This set consists of plates photographed from two perspectives using two different mobile devices - an iPhone (Apple Inc.) and an Android phone (Xiaomi Inc.). Hence, in some cases, there are two photographs of the same plate, but from two different perspectives using different mobile phones.

To facilitate the training of ML models we have split the dataset into train, validation and test holdouts at 0.7:0.2:0.1 ratio. Given the presence of the two perspectives, we ensured that the validation and test datasets had only one copy of the image to avoid validation data leaks. An example data point consisting of the input image, the well and plaque masks is shown in Fig. 1. The data deposit files are 3 zip-ed archives named VACVPlaque_train.zip, VACVPlaque_validation.zip and VACVPlaque_test.zip for the train, validation and test holdouts respectively. Each zip file contains 3 subdirectories, namely, “images”, “plaque_masks” and “well_masks”. The first subdirectory contains an original 8-bit RGB image of resolution 2448 x 3264 pixels (*H* x *W*). The latter folders contain images {*X*_*n*_} instance masks for plaques {${Y}_{n}^{1}$} and wells {${Y}_{n}^{2}$}.

## Technical Validation

To showcase that the VACVPlaque dataset is suitable for DL we have demonstrated it here. Manual quantification of virological plaque assay often requires ensuring that the phenotypes are visually distinct, circular and non-overlapping. Typically, these conditions are achieved by adjusting experimental settings such as the indicator cell line, virus concentration and time post-infection. Furthermore, for some viruses, it is common to add a semi-solid medium rather than a liquid to limit the cell-free spread of the virus.

In this work, we collated a dataset consisting exclusively of the Vaccinia Virus Western Reserve. However, given that phenotypically, researchers strive for similar phenotypic properties when assaying other viruses, our methods of detection should be applicable to other plaque assays provided that they adhere to our assumption of morphology and location respective to wells (in case of single-shot detection of plaques and wells). To validate this, we show experiments with changes in luminescence and zoom, which crudely emulate potential changes a different digital setup can introduce and discuss effects of such changes.

### Task and DL architecture design for dataset validation

To demonstrate that our dataset can be used for plaque assay quantification, we trained DL models for plaque and well detection using the input images and the ground truth (GT) we produced. We chose this task since it provides both detection and outlines of plaque phenotypes allowing for count and morphology analysis. Accompanied by instance masks of wells, the output of such a model could facilitate complete quantification of the phenotypes. This task of quantifying virological plaques in the dataset is the instance segmentation task commonly used in Computer Vision. This task allows us to both detect the individual plaques and measure their morphology by obtaining individual masks per each object. Furthermore, a similar task can be defined for the identification of individual wells. The intersection of the identified wells and plaques can provide information on the morphological properties of the plaque, as well as experimental conditions, which may differ between different wells.

**Fig. 3:**
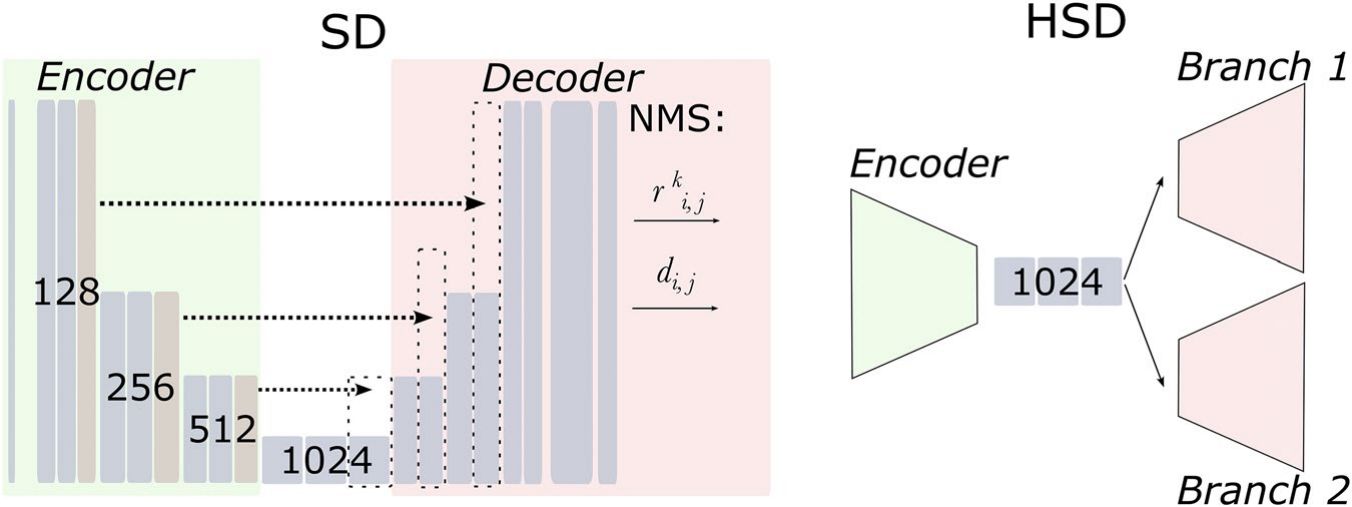
Architecture diagrams of the instance segmentation model for star-convex^21^ objects *StarDist (SD)*^34^ and a branched architecture *HydraStarDist (HSD)* based on it. *d*_*i*,*j*_ and ${r}_{i,j}^{k}$ refer to normalised Euclidean distance to the nearest background pixel and the Euclidean distance to the object boundary along *k* respectively (see Section Methods). Numbers stand for layer depth.

Given the highly circular shape of both plaques and wells, to address this task we have chosen the StarDist (SD)^34^ as the baseline. By design, SD enforces detected objects to be star-convex^21^, making it easier to generalise on small-to-medium-sized datasets. Segmentation of different objects such as plaques and wells would normally require performing it in two shots by two independent experiments. To circumvent this, we have proposed a modified version of the SD architecture, which we named HydraStarDist (*HSD*) (see Fig. 3). This architecture features a joint encoder and branched decoders for the two different yet nested objects. It penalises false predictions of the plaques outside the tissue culture plates implicitly. As seen in Figure 3, the HSD architecture is constructed in an analogous way to SD until the bottleneck i.e. (the narrowest part of the architecture). After the bottleneck, the decoder and the output are split the two branches.

### Model performance validation

**Fig. 4:**
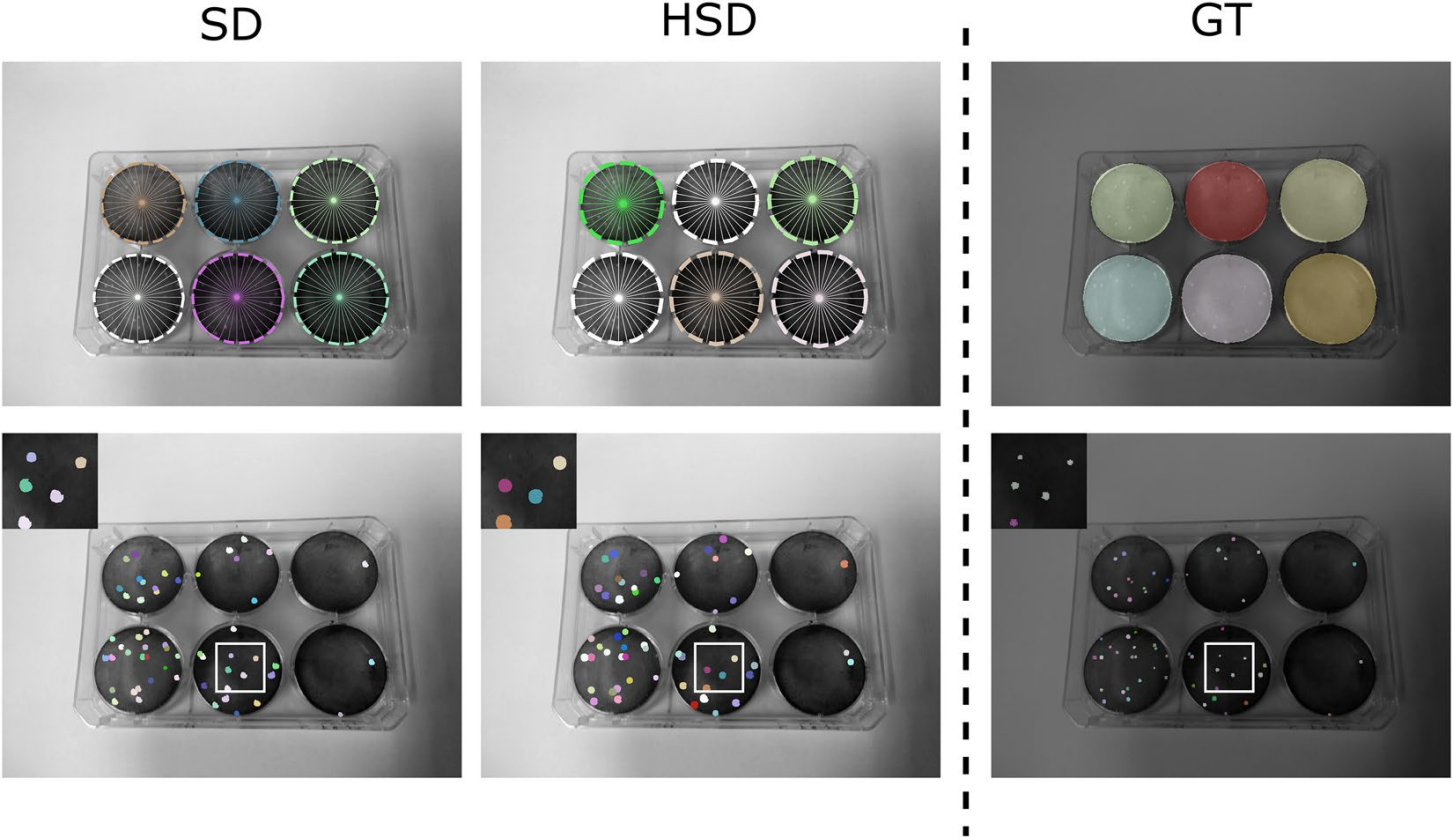
Instance segmentation results for *StarDist (SD)* and the branched architecture *HydraStarDist (HSD)* compared to the Ground Truth (GT). The well diameter is 35 mm for scale.

To verify the usability of the dataset to perform the task we described above, we trained and evaluated SD and HSD architectures on the VACVPlaque^14^ dataset. Results suggested that both architectures were able to segment individual wells and plaques quite effectively. Albeit, HSD missed some plaques, that SD managed to detect (Fig. 4, zoomed insets). Noteworthy, while SD required two dedicated models (two-shot) - one for plaques and one for wells, HSD segmented both kind of objects simultaneously (single-shot).

Next, to evaluate the performance quantitatively, we measured Intersection over Union over recalled objects (*IoU*_*R*_) and Average Precision (*AP*) (see Subsection Dataset validation performance evaluation metrics) from the StarDist paper^26^ (Tables 2 and 3). These metrics are commonly used for the evaluation of the instance segmentation task. Results suggest that the two-shot specialist SD outperforms the single-shot HSD for most *τ*’s for both *IoU*_*R*_ and *AP*. However, at least in the case of wells the differences in performance are negligible. Furthermore, even in the case of plaque objects at certain *τ*’s the performance of the HSD model is close to the performance of SD. These results suggest that not only is the VACVPlaque^14^ suitable for training an instance segmentation DL model, but also that this task is potentially attainable in a single-shot fashion using our dataset.

**Table 2:** Performance based on Intersection over Union over recalled objects (*IoU*_*R*_) (Eq. (5)) at different thresholds (*τ*).

Task	Architecture	*τ*								
		0.1	0.2	0.3	0.4	0.5	0.6	0.7	0.8	0.9
**Plaques**	*HSD* (ours)	0.420	0.418	0.410	0.392	0.358	0.282	0.170	0.066	0.009
	*SD*^34^	**0.494**	0.493	0.488	0.474	0.444	0.366	0.243	0.106	0.019
**Wells**	*HSD* (ours)	0.954	0.954	0.954	0.954	0.954	0.954	0.954	0.948	0.948
	*SD*^34^	**0.955**	**0.955**	**0.955**	**0.955**	**0.955**	**0.955**	**0.955**	**0.955**	0.944

In the table, *HSD*, *SD* represent the HydraStarDist, StarDist architectures respectively. **Best performance overall**. $\underline{\,{\rm{Best\; performance\; for\; specific}}\,\,\tau }$.

**Table 3:** Performance based on Average Precision (*AP*) (Eq. (6)) at different thresholds (*τ*).

Task	Architecture	*τ*								
		0.1	0.2	0.3	0.4	0.5	0.6	0.7	0.8	0.9
**Plaques**	*HSD* (ours)	0.618	0.604	0.561	0.495	0.407	0.271	0.135	0.045	0.005
	*SD*^34^	**0.730**	0.721	0.690	0.630	0.538	0.373	0.202	0.073	0.011
**Wells**	*HSD* (ours)	0.967	0.967	0.967	0.967	0.967	0.967	0.967	0.952	0.952
	*SD*^34^	**1.000**	**1.000**	**1.000**	**1.000**	**1.000**	**1.000**	**1.000**	**1.000**	0.974

In the table, *HSD*, *SD* represent the HydraStarDist, StarDist architectures respectively. **Best performance overall**. $\underline{\,{\rm{Best\; performance\; for\; specific}}\,\,\tau }$.

### Validation ablation study: luminescence and zoom impact

**Fig. 5:**
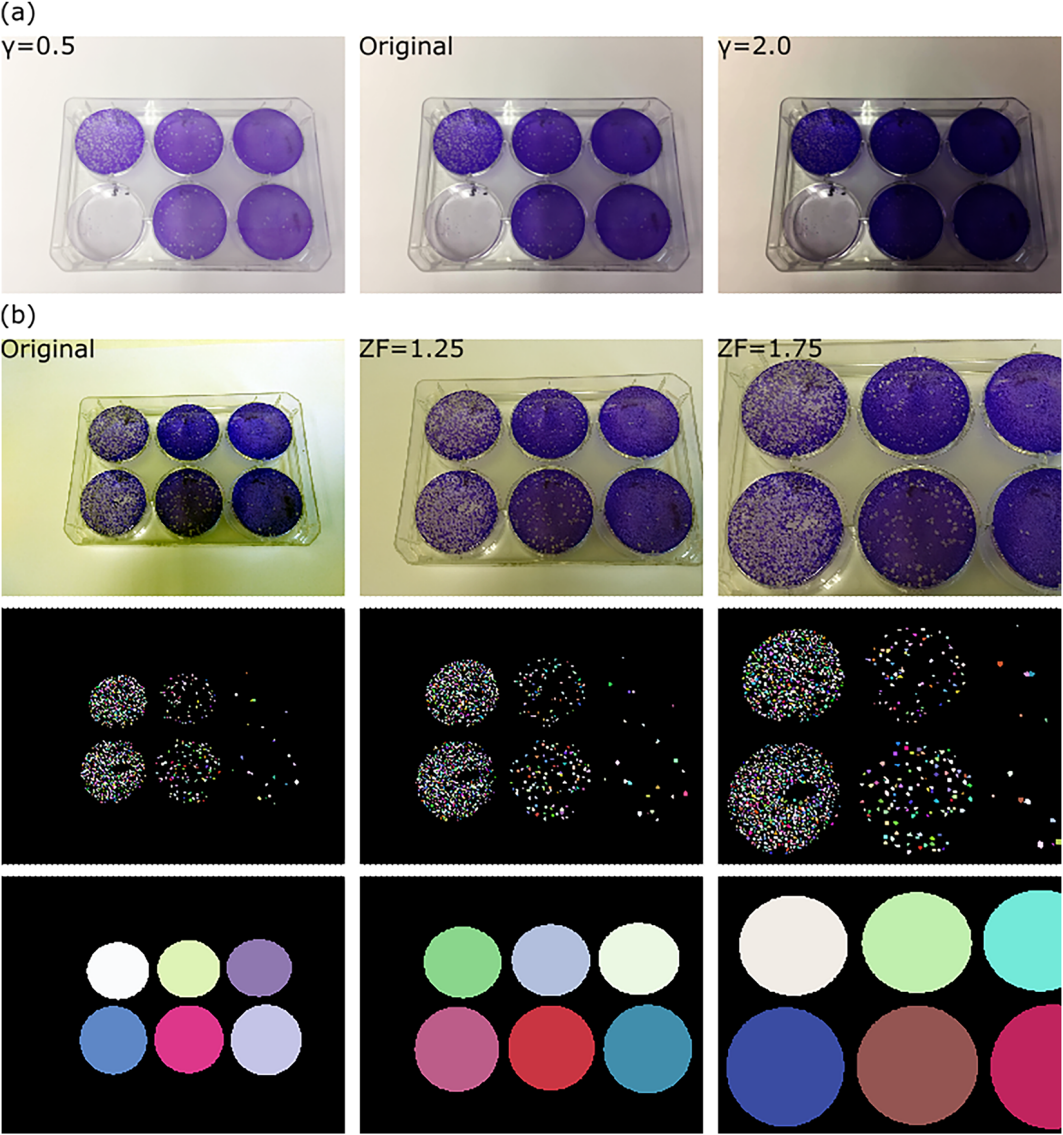
VACVPlaque^14^ ablation data examples containing **(a)** Plates at different luminescence levels for *γ* = 0.5, 2.0 (Eq. (7)) compared to the original (middle). **(b)** Plates at different zoom levels for *ZF* = 1.25, 1.75 (Eq. (8)) compared to the original (left) with corresponding plaque and wells masks in the corresponding column. The original well diameter is 35 mm for scale. Masks for panel **(a)** are omitted since change in luminescence does not affect the masks.

In this section we experiment with the impact of luminescence and zoom on digital photography images of our dataset. We achieve different levels of luminescence and zoom programmatically, using Gamma Correction^35^, (Eq. (7)) and different levels of zoom factor (Eq. (8)) respectively. The formula for luminescence change via Gamma Correction^35^ is given by,

7 $${I}_{x,y}^{{\prime} }={I}_{x,y}^{\gamma },$$

where *I*_*x*,*y*_ is the pixel value at the (*x*, *y*)^*th*^ position in the image *I* and ${I}^{{\prime} }$ is the resulting gamma-corrected image. The pixel values are first scaled between 0 and 1. We have used the inbuilt Python function https://scikit-image.org/docs/stable/api/skimage.exposure.html#skimage.exposure.adjust_gamma inside the Scikit-Image package for this. We set *γ* to 0.5 and 2 in our experiments as shown in Tables 4, 5 and Fig. 5.

**Table 4:** Performance based on *IoU*_*R*_ at different luminescence levels at difference values of *γ* (Eq. (7)) and at different thresholds (*τ*).

Task	*γ*	Architecture	*τ*								
			0.1	0.2	0.3	0.4	0.5	0.6	0.7	0.8	0.9
**Plaques**	0.5	*HSD* (ours)	0.433	0.432	0.424	0.408	0.376	0.304	0.190	0.076	0.012
		*SD*^34^	**0.496**	0.495	0.490	0.475	0.445	0.366	0.246	0.108	0.019
	2	*HSD* (ours)	0.385	0.384	0.378	0.363	0.334	0.266	0.161	0.061	0.010
		*SD*^34^	**0.493**	0.492	0.487	0.472	0.442	0.364	0.241	0.105	0.020
**Wells**	0.5	*HSD* (ours)	**0.957**	**0.957**	**0.957**	**0.957**	**0.957**	**0.957**	**0.957**	**0.957**	0.950
		*SD*^34^	0.944	0.944	0.944	0.944	0.944	0.944	0.944	0.932	0.897
	2	*HSD* (ours)	**0.958**	**0.958**	**0.958**	**0.958**	**0.958**	**0.958**	**0.958**	0.952	0.945
		*SD*^34^	0.955	0.955	0.955	0.955	0.955	0.955	0.955	0.955	0.928

In the table, *HSD*, *SD* represent the HydraStarDist, StarDist architectures respectively. **Best performance overall**. $\underline{\,{\rm{Best\; performance\; for\; specific}}\,\,\tau }$.

**Table 5:** Performance based on *AP* at different luminescence levels at difference values of *γ* (Eq. (7)) and at different thresholds (*τ*).

Task	*γ*	Architecture	*τ*								
			0.1	0.2	0.3	0.4	0.5	0.6	0.7	0.8	0.9
**Plaques**	0.5	*HSD* (ours)	0.639	0.623	0.580	0.521	0.433	0.298	0.153	0.052	0.007
		*SD*^34^	**0.730**	0.721	0.687	0.622	0.531	0.368	0.202	0.074	0.011
	2	*HSD* (ours)	0.570	0.558	0.526	0.471	0.394	0.267	0.134	0.043	0.006
		*SD*^34^	**0.728**	0.721	0.689	0.624	0.533	0.369	0.199	0.072	0.012
**Wells**	0.5	*HSD* (ours)	0.977	0.977	0.977	0.977	0.977	0.977	0.977	0.977	0.962
		*SD*^34^	**0.984**	**0.984**	**0.984**	**0.984**	**0.984**	**0.984**	**0.984**	0.954	0.881
	2	*HSD* (ours)	0.940	0.940	0.940	0.940	0.940	0.940	0.940	0.923	0.912
		*SD*^34^	**1.000**	**1.000**	**1.000**	**1.000**	**1.000**	**1.000**	**1.000**	**1.000**	0.938

In the table, *HSD*, *SD* represent the HydraStarDist, StarDist architectures respectively. **Best performance overall**. $\underline{\,{\rm{Best\; performance\; for\; specific}}\,\,\tau }$.

For studying the effect of change of zoom, we have used the following formula followed by resizing to dimensions of images used for original experiments. The zoomed images are obtained by,

8 $${I}^{{\prime\prime} }=I[(\frac{W}{2}-\frac{W}{2ZF},\frac{H}{2}-\frac{H}{2ZF}),(\frac{W}{2}+\frac{W}{2ZF},\frac{H}{2}+\frac{H}{2ZF})],$$

where *I*^*″*^ is the zoomed image and *ZF* is what we call the Zoom Factor that we set to 1.25 and 1.75 in our experiments as shown in Tables 6, 7 and Fig. 5.

**Table 6:** Performance based on *IoU*_*R*_ at different zoom levels (Eq. (8)) and at different thresholds (*τ*).

Task	ZF	Architecture	*τ*								
			0.1	0.2	0.3	0.4	0.5	0.6	0.7	0.8	0.9
**Plaques**	1.25	*HSD* (ours)	0.456	0.455	0.448	0.431	0.394	0.310	0.183	0.065	0.007
		*SD*^34^	**0.538**	0.537	0.533	0.522	0.494	0.414	0.278	0.117	0.018
	1.75	*HSD* (ours)	0.450	0.450	0.446	0.432	0.403	0.333	0.214	0.077	0.006
		*SD*^34^	**0.540**	0.539	0.537	0.527	0.505	0.440	0.310	0.136	0.012
**Wells**	1.25	*HSD* (ours)	**0.934**	**0.934**	**0.934**	**0.934**	**0.934**	**0.934**	0.929	0.929	0.901
		*SD*^34^	0.933	0.933	0.933	0.933	0.933	0.933	0.933	0.933	0.906
	1.75	*HSD* (ours)	0.734	0.734	0.734	0.729	0.704	0.645	0.520	0.322	0.080
		*SD*^34^	**0.796**	**0.796**	**0.796**	**0.796**	0.788	0.779	0.674	0.501	0.182

In the table, *HSD*, *SD* represent the HydraStarDist, StarDist architectures respectively. **Best performance overall**. $\underline{\,{\rm{Best\; performance\; for\; specific}}\,\,\tau }$.

**Table 7:** Performance based on *AP* at different zoom levels (Eq. (8)) and at different thresholds (*τ*).

Task	ZF	Architecture	*τ*								
			0.1	0.2	0.3	0.4	0.5	0.6	0.7	0.8	0.9
**Plaques**	1.25	*HSD* (ours)	0.629	0.618	0.581	0.520	0.429	0.284	0.138	0.041	0.004
		*SD*^34^	**0.757**	0.750	0.726	0.676	0.589	0.417	0.227	0.078	0.010
	1.75	*HSD* (ours)	0.614	0.610	0.587	0.538	0.460	0.328	0.174	0.052	0.003
		*SD*^34^	**0.731**	0.728	0.712	0.671	0.602	0.456	0.263	0.093	0.007
**Wells**	1.25	*HSD* (ours)	0.969	0.969	0.969	0.969	0.969	0.969	0.954	0.954	0.896
		*SD*^34^	**0.992**	**0.992**	**0.992**	**0.992**	**0.992**	**0.992**	**0.992**	**0.992**	0.931
	1.75	*HSD* (ours)	0.933	0.933	0.933	0.905	0.813	0.662	0.442	0.220	0.044
		*SD*^34^	**0.977**	**0.977**	**0.977**	**0.977**	0.946	0.917	0.664	0.398	0.110

In the table, *HSD*, *SD* represent the HydraStarDist, StarDist architectures respectively. **Best performance overall**. $\underline{\,{\rm{Best\; performance\; for\; specific}}\,\,\tau }$.

A visual effect of the change in these aspects are shown in Fig. 5. The performance under changes in luminescence and zoom can be observed from the *AP* and *IoU*_*R*_ values in Tables 4, 5, 6 and 7. As we can see from these tables, the results follow similar patterns as the results in Tables 2 and 3 with the exception that in Table 4 for wells, and in Table 6 for ZF equals 1.25 for wells, the performance of HSD improves for both lower and higher luminescence as compared to SD under the same conditions. Other changes in scores between the original and ablation study tables are too nuanced to claim as the effect of luminescence or zoom changes alone, therefore, the performance under ablation conditions are fairly stable and similar to that under original conditions. Lastly, it is important to note that we have not experimented with a combination of both effects (luminescence and zoom) and only treat them as individual effects.

Despite its long history, the plaque assay remains as important for the quantification of infectious virus today as it was almost a century ago^8^. Yet, with the advent of Computer Vision, ML and DL our approaches to the quantification of images have significantly improved in the meantime. In this work, we present an open dataset of VACV plaque assays photographed with a mobile phone, allowing us to leverage these advances. Furthermore, we demonstrate how a state-of-the-art DL architecture could address this task. Finally, we propose an improvement on the state-of-the-art architecture, allowing us to address the quantification task in an end-to-end manner using the VACVPlaque dataset presented here.

## Data Availability

All newly contributed code is available on GitHub at https://github.com/casus/VACVPlaque under the BSD 3-Clause License.
